# The Book World of Medicine and Science

**Published:** 1903-08-01

**Authors:** 


### The Book World of Medicine and Science.

Death and Sudden Death. By P. Brouardel and F. L. Benham, M.D. (London: Baillière, Tindall and Cox. 1902. Pp 330, 8vo. Price 10s. 6d. net.)

Death and Sudden Death is, in spite of its subject, one of the most fascinating of medical works. Its form is that of lectures, on the Signs of Death and on Sudden Death, as delivered by Professor Brouardel, of Paris, to students of the Faculty of Medicine. It has been translated by Dr. Lucas Benham, who has, in this the second edition, made such additions and amplifications as have seemed called for. Dr. Benham deserves congratulations on the way he has performed his task; he has made the translation with rare taste and aptness, and has well preserved the Gallic brilliancy and logic of the original. We do not hesitate to say that the work should be read by every practitioner, and that it should be constantly referred to by all who have to conduct necropsies. It is an extraordinary reflection that while the study of the phenomena attending birth has been laboured to a degree, yet, in England at any rate, the terminal phenomena of life have been hardly studied seriously. The public are still satisfied with death certificates given without investigation, and coroners and their juries appreciate nothing so much as "evidence showing that death resulted from stoppage of the heart's action." A study of Brouardel's illuminating and philosophic treatise should convince anyone that we are still too ready to content ourselves with phrases and formulæ which carry conviction solely through repetition. Brouardel cuts away the ground from beneath the feet of those who so often speak of death as due to syncope, and, with Brown Séquard, he develops the facts of "inhibition," asking whether in many cases individuals in state of apparent death might not return to life if it were recognised that the blood was retaining vital properties and that respiration and cardiac action had ceased through "inhibition." The phenomena of rigor mortis, post-mortem circulation, and putrefaction are admirably discussed, and a vivid picture is drawn of the processes revealed to us by Mégnin's classical researches on the "entomology of putrefaction." A chapter on the legislation which has regard to death certification and kindred subjects completes the first portion of the work. The second portion deals, as we have said, with sudden death, and is particularly instructive. As Brouardel says, all death is literally sudden; what is called sudden death is "unforeseen" death—death not preceded by alarming or warning phenomena. And in a relatively large number of cases (say from 8 to 10 per cent.) no autopsy, however minute or painstaking, can actually determine the cause of death. Perhaps all that can be said is that no traces of poison, no marks of violence can be found. In such cases the duty of the expert is to state merely what he finds, and, in answer to the inquiry, "what caused death?" to say, "I cannot tell; the evidence is not forthcoming." This is in reality the most dignified course to take; the unscientific course is to talk vaguely of "stoppage of the heart's action," which, after all, comes peculiarly near the classical remark of death being due to "cessation of the vital functions." This second part of Brouardel's work contains a wealth of pathological information from which we would fain cull instances. But it must suffice to allude to the lucidity with which kidney disease is demonstrated to be the determining cause of many sudden deaths, and to the remarkable series of cases given in which death has resulted from reflex inhibition, as, for instance, in making simple vaginal examinations. In conclusion we again commend the patient study of this book to every practitioner and medico-legal expert.

Diseases of the Skin: an Outline of the Principles and Practice of Dermatology. By Malcolm Morris, Consulting Surgeon to the Skin Department, St. Mary's Hospital, etc. (Cassell and Company, Limited, London, Paris, New York, and Melbourne. 1903. New Edition. Octavo, pp. 642, 2 Coloured Plates and 58 Plain Figures.)

It is not too much to say that, whereas diseases of the skin are among the easiest to demonstrate, they are, above all others, the most difficult to describe. The distinctions between one affection and another which are so obvious when they are pointed out upon the patient's body appear insignificant when they are put down in black and white, and, in the case of many treatises on the skin, the reader may be excused if he should fail to fully appreciate the correct picture of the diseases which are described therein. Mr. Malcolm Morris, however, possesses the power of lucid description in a high degree, and his book, from its first appearance, became indispensable to the student and the practitioner. His method of following the full description of a disease by a summary of its chief points and of contrasting these with the salient features of other diseases for which it might be mistaken is admirable, and leaves no excuse for want of comprehension on the part of the reader. Another most praiseworthy point about the book is the thoroughness with which all methods of treatment are described. There are, it is true, some small slips but these do not detract from the real value of the book. For instance, the rash of scarlet fever is said to last for 10 or 12 days, a period which would be exceptionally long. Again, on p. 96 it is said that anæmia is a pronounced feature of the tubercular diathesis, which is incorrect if we take the exact condition of the blood as the test of the presence of anæmia. Among omissions, we failed to find any description of the so-called enema rash. We think, further, that Mr. Morris is, perhaps, rather too fond of the term angio-neurosis. He seems to apply this term even to inflammations which are caused by poisons circulating in the blood; and he has gone so far as to place purpura among the angio-neuroses, in spite of his own statement to the effect that it may be due to over-distension of the blood-vessels, changes in the blood, etc. Much of the pathology in the book is, however, excellent, but the microphotographs constitute a blemish. Most of them show little more than a rough outline, and they could be of no great use to anyone who did not know enough morbid histology to make out what they were intended to represent. The other pictures in the book are good.

